# Long-term multichannel recordings in *Drosophila* flies reveal altered predictive processing during sleep compared with wake

**DOI:** 10.1242/jeb.250165

**Published:** 2025-06-03

**Authors:** Matthew N. Van De Poll, Bruno van Swinderen

**Affiliations:** Queensland Brain Institute, The University of Queensland, St Lucia, QLD 4072, Australia

**Keywords:** Vision, Electrophysiology, Sleep, Visual responsiveness

## Abstract

During sleep, behavioral responsiveness to external stimuli is decreased. This classical definition of sleep has been applied effectively across the animal kingdom to identify this common behavioral state in a growing list of creatures, from mammals to invertebrates. Yet, it remains unclear whether decreased behavioral responsiveness during sleep is necessarily associated with decreased responsiveness in brain activity, especially in insects. Here, we performed long-term multichannel electrophysiology in tethered *Drosophila melanogaster* flies exposed continuously to repetitive visual stimuli. Flies were still able to sleep under these visual stimulation conditions, as determined by traditional immobility duration criteria for the field. Interestingly, we did not find any difference between responses to repetitive visual stimuli during sleep compared with wake when we recorded local field potentials (LFPs) across a transect of the fly brain from optic lobes to the central brain. However, we did find LFP responses to be altered when visual stimuli were variable and of lower probability, especially in the central brain. Central brain responses to less predictable or ‘deviant’ stimuli were lower during the deepest stage of sleep, a time of quiescence characterized by more regular proboscis extensions. This shows that the sleeping fly brain processes low-probability visual stimuli in a different way from more repeated stimuli, and presents *Drosophila* as a promising model for studying the potential role of sleep in regulating predictive processing.

## Teaser

**Highlighted Article:** The sleeping fly brain still responds to visual stimuli; however, response amplitudes depend on stimulus history and probability, as well as recording location.

## INTRODUCTION

The key criterion identifying sleep in an animal is decreased responsiveness compared with wake (Campbell and Tobler, 1984; Kales and Rechtschaffen, 1968). Typically, this is investigated in animals by measuring behavioral responsiveness to probing stimuli, such as mechanical vibrations, loud noises or light flashes. Responsiveness during sleep can also be tracked in brain activity recordings, where event-related potentials in response to applied stimuli are typically lower or even abolished during sleep compared with wake. Even while peripheral sensory processing continues, as in the case of auditory stimuli in many animals, sensory perception can be attenuated during sleep. For example, brain responses to certain auditory stimuli are decreased during sleep in human electroencephalography (EEG) recordings, revealing impaired predictive processing of unexpected sounds during specific sleep stages (Strauss et al., 2015; Andrillon and Kouider, 2020). This suggests that a key feature of the sleeping brain is a decreased ability to register changes in the external environment. Whether this is true in all animals that sleep is unclear, although there is also evidence that sleeping animal brains are keeping track of variations in environmental stimuli. Sleeping mice recorded with EEG showed stronger brain responses to conditioned aversive stimuli than to neutral stimuli during both rapid eye movement (REM) and non-REM (NREM) sleep (van Kronenberg et al., 2022). Similarly, a behavioral study in *Drosophila melanogaster* found that sleeping flies can process and respond to different odors, with ethologically relevant odors more likely to wake flies up (French et al., 2021). This suggests a level of sensory processing in the sleeping fly brain.

The fact that flies sleep is now well established (Shafer and Keene, 2021) and work over the past decade has shown that, like other animals, fly sleep is also characterized by distinct stages (van Alphen et al., 2013; Yap et al., 2017; Wiggin et al., 2020; Tainton-Heap et al., 2021; van Alphen et al., 2021; Jagannathan et al., 2024; Joyce et al., 2024). These stages appear to comprise an ‘active’ phase when the fly brain seems as active as during wakefulness (but behaviorally the flies are quiescent and arousal is low; Tainton-Heap et al., 2021; Anthoney et al., 2023) and a ‘quiet’ phase when brain activity is decreased and when flies display characteristic microbehaviors such as rhythmic proboscis extensions (van Alphen et al., 2021; Jagannathan et al., 2024). Evidence for altered brain activity during sleep in flies was originally drawn from single-channel local field potential (LFP) recordings, which allowed for long-term recordings in behaving flies (Nitz et al., 2002). In a recent study, we achieved long-term recordings in behaving (and sleeping) flies implanted with a 16-channel silicone probe, which provided insight into how different regions of the fly brain behave across changes in behavioral state (Jagannathan et al., 2024). These experiments confirmed distinct sleep stages in flies, but surprisingly showed that the entire fly brain sleeps, including the outer optic lobes. How visual stimuli might be processed from the optic lobes to the central brain during sleep is unknown.

Unlike humans and many other animals, flies and other insects cannot shut their eyes when they sleep. Yet, visual responsiveness is suppressed in sleeping insects, as shown in behavioral studies (Campbell and Tobler, 1984) as well as in electrophysiological recordings (Kaiser and Steiner-Kaiser, 1983; van Swinderen and Greenspan, 2003). This suggests a central arousal mechanism designed to block certain visual stimuli during sleep, although it is not known how this mechanism might work or what parts of the insect brain might be involved. One obvious candidate is the central complex, a region in the *Drosophila* brain associated with perception and decision making (Wolff et al., 2015; Sun et al., 2017; Lyu et al., 2022) that exhibits alterations in stimulus responses during sleep (Tainton-Heap et al., 2021; Troup et al., 2018). In the present study, we adapted a long-term multichannel recording preparation (Jagannathan et al., 2024) for *Drosophila* flies exposed to repetitive visual stimuli to investigate whether visual responsiveness across the fly brain changes during sleep. We first examined the effect of steady-state visually evoked potentials (SSVEPs; Norcia et al., 2015), as done for previous work in awake (Paulk et al., 2013b) and anesthetized (Cohen et al., 2016) flies. SSVEPs are oscillatory LFPs that are evoked by repetitive visual stimuli (Vialatte et al., 2010). In humans, SSVEPs have been used to study visual perception, as the amplitude of these responses was found to be modulated by attentional states (Norton et al., 2017; Norcia et al., 2015). Repetitive visual stimuli also evoke SSVEPs throughout the fly brain (Paulk et al., 2013b, 2015; Cohen et al., 2016), and SSVEPs recorded from the central fly brain appear to be modulated by attention-like states (van Swinderen, 2012; Grabowska et al., 2020). In the current study, we manipulated the probability of repetitive visual stimuli to investigate the effect on SSVEP responses in the fly brain, and asked whether visual predictive processing was altered during spontaneous sleep compared with wake.

## MATERIALS AND METHODS

### Animals

To maintain genetic consistency, all animals used in experiments were from a cross of a R23E10-GAL4 driver line (cs; P{y[+t7.7] w[+mC]=GMR23E10-GAL4}attP2) with a UAS-Chrimson line (w[1118]; P{y[+t7.7] w[+mC]=20XUAS-CsChrimson.mCherry}su(Hw)attP5). CsChrimson was included in the cross to maintain consistency with flies from other experiments but was never activated. Animals were raised at 25°C on standard cornmeal medium. Female non-virgin F1 flies were collected between 3 and 9 days post-eclosion and used for experiments.

### Tethering

Tethering was performed as described previously (Van De Poll and van Swinderen, 2024). In brief, animals were cold-anesthetized at 2°C and glued dorsally to a short tungsten rod. A silver ground wire was also inserted gently into the thorax. After tethering, the animals were placed atop an air-supported ball as previously described (Jagannathan et al., 2024).

### Experimental setup

While walking on an air-supported ball, animals were able to view a custom-built matrix of 90 interleaved blue and green LEDs (45 of each color), positioned to the front–left and level with the fly. The intensity of both green and blue LEDs was calibrated to be 0.005 mW mm^−2^ at the eye with a photometer.

A 16 channel multielectrode (NeuroNexus A1×16-3mm 50-177) was inserted into the ipsilateral eye to the LED panel, feeding through a preamplifier (Tucker-Davis Technologies RA16PA) into a data acquisition base station (Tucker-Davis Technologies RZ5).

During experiments, a camera placed lateral to the fly recorded behavioral activity, including movements of the legs and abdomen, as well as the proboscis. A custom Python script acquired and saved the video data as well as time stamps for each frame (Jagannathan et al., 2024).

Stimulus paradigms were coded on a computer running Windows 10 (Dell Precision 7910), and then sent to the base station, which controlled the transmission of the signal to the power circuit controlling the LEDs (custom-built).

Room lighting was provided according to a 12 h:12 h On/Off cycle, switching on at 07:00 h and off at 19:00 h.

### Stimuli

Stimuli consisted of full-field flashes (all LEDs of a given color active) of the LED panel at a fixed 10 Hz interval, with a 50% duty cycle. Each experiment comprised a number of ‘trials’, where each trial was defined as a 20 s period of stimuli (200 stimulus events). The stimulus signal was a square wave, such that the LEDs moved between the On and Off states with no ramping.

The arrangement of stimulus events within each trial could be one of three combinations, depending on each experiment: ‘Carrier only’ trials consisted of a unitary color (e.g. all green), at the F1 frequency of 10 Hz. ‘Phasic deviant’ trials contained a deviation in color (e.g. green deviant amongst blue carriers) at a rate of 2 Hz, such that every fifth stimulus event was a deviant. ‘Jittering deviant’ trials were similar, except that the position of the deviants in the sequence was shifted according to a normal distribution centered over each original deviant sequence position (i.e. most likely to be 5th element, less likely to be 4th or 6th element, etc.), which resulted in a mean (±s.e.m.) number of 3.94±2.74 carriers between each deviant stimulus. In effect, this meant that most times after a deviant stimulus there would be four carrier stimuli before the next deviant, but sometimes there might only be three carriers, or a longer gap of five carriers, and so on. Color assignations for 20 s stimulus trials were counterbalanced such that each color was a carrier/deviant for an equal number of trials. For comparisons between carriers and deviants of a particular color (e.g. green), we utilized the counterbalanced nature of stimulus trials to only make within-color comparisons. Thus, as the local color history of a green deviant is ‘blue → green’ we compared these deviant events against green carriers from stimulus trials where they were preceded by a blue deviant, and vice versa for blue deviants.

### Experiment durations

For initial experiments during daytime hours, the duration was around 1 h per fly (72 trials). In the later experiments incorporating sleep analysis, the experiments ran for a portion of the day and a full night cycle, typically around 16 h (2450 trials). As in a previous study using the same recording preparation (Jagannathan et al., 2024), only the first 6 h of each recordings was used, based on qualitative observation of signal quality over time for the compiled dataset. Also following our previous study (Jagannathan et al., 2024), we excluded flies whose LFP activity failed to show a visual response during the calibration portion of the experiment. Additionally, flies that were behaviorally inactive from the outset of electrode insertion and the majority of the calibration period (∼10 min) were also not continued for long-term recordings.

### Data

**Fig. 1.**
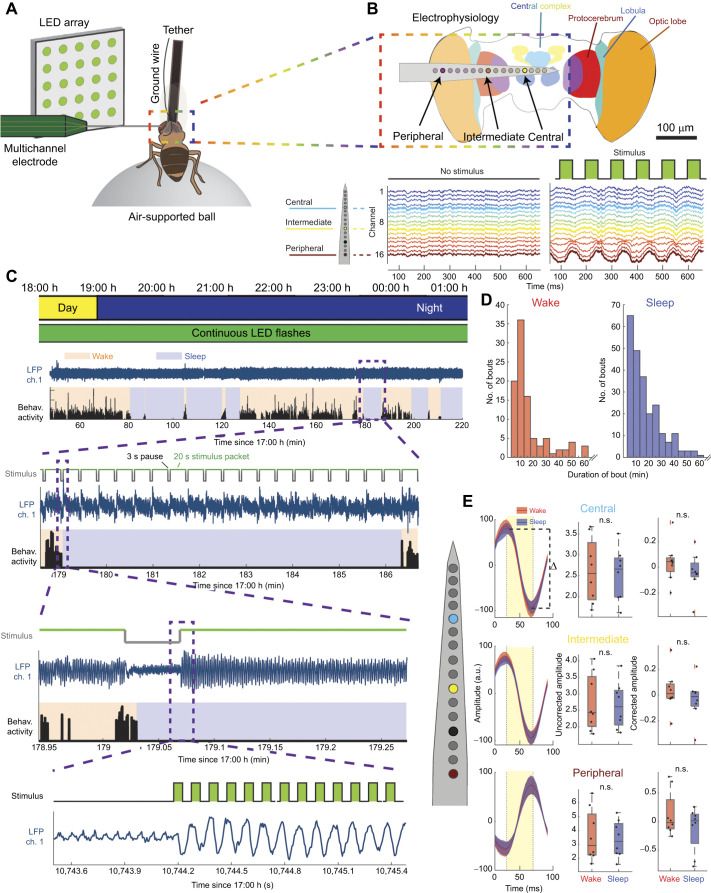
**Visually evoked local field potential activity across the fly brain during wake and sleep.** (A) Schema of a tethered fly positioned on an air-supported ball. An LED array projects visual stimuli to the left eye, which also has a 16-channel multi-electrode inserted. (B) Top: schematized fly brain, showing the approximate position of electrode channels neuroanatomically. Marked are exemplar central, intermediate and peripheral channels, showing their typical positions during a recording. Bottom: example local field potential (LFP) activity from an individual fly during an unstimulated recording period (left) and a period with a 10 Hz visual stimulus (right). (C) Top: schematized representation of recording occurring across a day/night cycle. Underneath, the presence of stimuli across time is represented. Second from top: example data from an early portion of an individual recording. Shown is LFP activity from an example channel (blue), as well as fly movement activity (black). Shading indicates periods when the fly was determined to be awake (orange) or sleeping (blue), based on the movement activity. Third from top: subset of the above, from a span of around 7 min, showing the time course of stimulus trials (top, gray/green trace, where green represents periods of stimulus delivery), LFP activity from an example channel (middle, blue) and movement activity (bottom, black). Fourth from top: expanded view of the above activity, showing a transition from wake to sleep, as well as the onset of a stimulus bout. Bottom: a 2 s period of the above, showing stimulus-evoked activity in a central channel. The top trace (‘Stimulus’) represents stimulus event onset and offset timing. (D) Distribution of bout durations for wake (left, red) and sleep (right, blue) (*N*=8 flies). (E) Left: averaged LFP (*N*=8) in response to visual stimulus presentation during wake (red) and sleep (blue). Stimulus time course is displayed with yellow shading. Dashed line indicates example calculation of total distance between peak and trough of an averaged event-related potential (‘Δ’), herein referred to as ‘amplitude’. Top, middle and bottom respectively relate to LFP activity in central, intermediate and peripheral channels. Middle: uncorrected amplitude between peak and trough of average LFP during wake and sleep (n.s., one-way ANOVA with Bonferroni correction; *N*=8). Right: mean-corrected amplitude of LFP amplitudes during wake and sleep (n.s., not significant; one-way ANOVA with Bonferroni correction). Dots represent individual fly data. Box plots show median, upper and lower quartiles and 1.5× interquartile range.

**Fig. 2.**
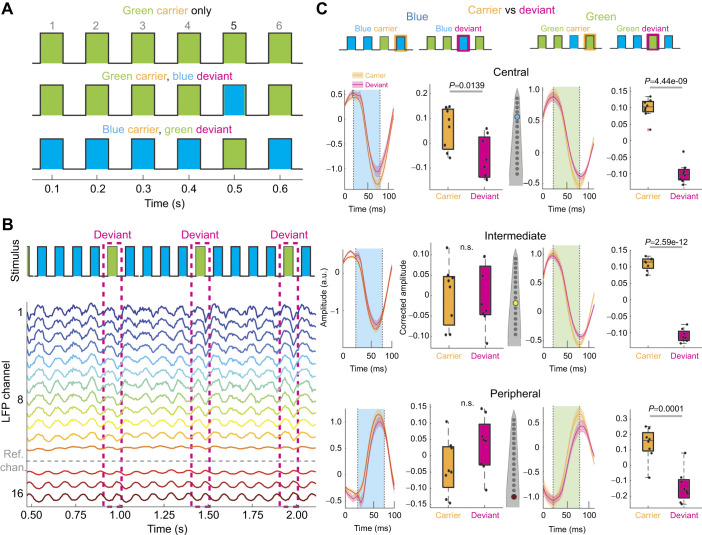
**Decreased responsiveness to deviant stimuli.** (A) Schematized stimulus trials, showing periods with a continuously repeated carrier (top) and those with color deviants (middle and bottom). Numbers above schema represent position in the sequence of events. All conditions are counterbalanced for color across individuals. (B) Stimuli and corresponding LFP activity across the fly brain during a portion of a stimulus block, showing stimulus event identity and matching LFP. Deviant events are highlighted with a dashed pink box. Gray dashed line indicates the polarity reversal channel for this fly, which was used for as a reference channel for noise subtraction. (C) Left: LFPs and corrected amplitudes across the channel in response to a blue carrier (orange shading) or blue deviant (pink shading). Right: as for left, but for green carriers/deviants. Dots represent individual fly data. Box plots show median, upper and lower quartiles and 1.5× interquartile range. *P*-values where indicated represent one-way ANOVA with Bonferroni correction (*N*=8).

LFP data were acquired from the inserted multichannel electrode at 24 kHz, downsampled after experiments to either 1000 Hz or 200 Hz for 1 h and 16 h recordings, respectively. Re-referencing of the LFP data was performed by subtracting the data of one channel from the remaining 15 channels. Selection of the channel to subtract was based on identifying the closest channel around which the polarity of visually evoked responses inverted, which by definition also had the smallest response amplitudes (see Fig. 1B, bottom; Fig. 2B). In addition to the 16 LFP channels, these data contained time-synchronized signals for stimulus identity, as well as stimulus color and trial state.

Time stamps were used to synchronize the LFP and behavioral video recordings, to allow for separation of LFP data by activity state and other factors.

Data are available from the Dryad digital repository (https://doi.org/10.5061/dryad.7pvmcvf5h); code is available from Github (https://github.com/MNVDP/MultichannelLFPAnalysis).

### Statistics

All data analysis was performed with custom MATLAB (R2020a) scripts. LFP data were downsampled as mentioned and then line noise was removed at 50 Hz multiples. Most data analysis was performed on the SSVEP evoked by each stimulus event, with the primary metric being the change in amplitude between the highest and lowest points of the SSVEP, following the stimulus onset. Normalization was performed within channel by calculating the mean amplitude of all stimulus events for each color (e.g. all green stimuli, regardless of identity) and expressing each individual event as a proportion of that value.

To allow us to compare across conditions during comparisons, we calculated the average across groups (within flies) and then subtracted this from individuals, thus yielding a comparison-specific, normalized value, referred to herein as ‘corrected amplitude’. For example, in comparing green deviants against carriers, the mean of the deviant and carrier response for each fly would be calculated and then subtracted from the two groups, thus expressing any change as a relative value.

## RESULTS

### Recording visual responses across the fly brain during sleep and wake

Tethered female *Drosophila* flies (3–9 days old) were able to walk for hours at a time on an air-supported ball while being presented with flashing visual stimuli (Fig. 1A). During this time, LFP activity was recorded from 16 different locations across their brain, spanning from the left retina to the central brain (Fig. 1B, top). In the calibration period prior to the presentation of visual stimuli, LFP recordings across the fly brain revealed a characteristic profile, with synchronized endogenous activity across the central brain through to the optic lobe (Fig. 1B, bottom left). Following this, as shown in previous studies using a similar multichannel preparation (Paulk et al., 2013b), the fly brain responded to periodically flashing visual stimuli with synchronized evoked potentials (Fig. 1B, bottom right). A polarity reversal in the LFP signal was evident in the vicinity of the lobula (around electrodes 11–14), and this electrophysiological feature was used to ensure reproducible electrode insertions across flies, and to serve as a reference channel (see Materials and Methods). As in our previous study (Jagannathan et al., 2024), we partitioned data into three separate brain regions: central brain (inboard of the polarity reversal by 8 channels, typically around channel 3), intermediate (inboard by 3 channels, typically around channel 8) and peripheral (outboard by 3 channels, typically around channel 14).

Brain recordings were performed over several hours, spanning the natural transition to night-time sleep (Jagannathan et al., 2024; see Materials and Methods). Visual stimuli were delivered via an array of green and blue light emitting diodes (LEDs) positioned 16 cm in front of the fly, slightly to the left and thus ipsilateral to the recorded brain hemisphere (Fig. 1A). Visual stimuli were presented throughout the experiment at 10 Hz (50 ms duty cycle), in packets of 20 s, alternating with 3 s pauses (Fig. 1C). The stimuli produced robust SSVEPs throughout the fly brain (responses in the central brain are shown in Fig. 1C, bottom panel). We examined the behavioral data for evidence of sleep, defining sleep as periods of behavioral quiescence lasting 5 min or more (see Materials and Methods). Remarkably, flies were able to sleep while presented with this continuously flickering visual stimulus, displaying median sleep bout durations of 12.8 min and wake bout durations of 11.2 min (Fig. 1D; an example sleep bout flanked by waking activity is shown in Fig. 1C, second panel from the top). These sleep/wake bout durations are comparable with durations found in previous work utilizing the same multichannel LFP setup without visual stimulation (Jagannathan et al., 2024). Notably, we found no significant difference in the average SSVEP amplitude during sleep compared with wake, for any of the three broadly defined brain regions (Fig. 1E), even after correcting for individual fly SSVEP amplitude variability (Fig. 1E, right column; see Materials and Methods). This suggests that behavioral state does not modulate the average LFP response to the repetitive visual stimuli in this paradigm.

### Decreased responsiveness to deviant stimuli

We next questioned whether the probability of a stimulus occurring might affect the response across the fly brain. In the preceding experiment, the probability of a 50 ms green flash occurring within the 20 s visual sessions was 100%. To test whether stimulus probability altered visual responsiveness, we introduced a lower probability ‘deviant’ stimulus of a different color within the train of 10 Hz stimuli (Fig. 2A). Thus, a blue deviant stimulus was presented after every four green stimuli, such that the green ‘carrier’ stimuli still occurred at 10 Hz while the blue deviant stimuli occurred at 2 Hz. The experiment was counterbalanced so that for some 20 s sessions the blue stimuli were deviant, while in other sessions the green stimuli were deviant. This arrangement allowed us to compare the responses to carriers and deviants strictly within a color, to potentially account for any differences in color processing. Thus, we asked whether the LFP response to a green or blue deviant occurring at 2 Hz was different from the response to a green or blue carrier occurring at 10 Hz. To ensure that our comparisons were historically identical and only contingent on the stimulus probability, we specifically compared carriers preceded by a deviant with (identically colored) deviants preceded by a carrier (because both conditions involved a color change). We first only examined responses during waking epochs, to determine whether responses varied in an aroused brain. An example of LFP responses across the brain to carrier and deviant stimuli is shown in Fig. 2B. We found that stimulus probability had a significant effect on SSVEP amplitude: in the central brain, responses to deviant stimuli were smaller than responses to carrier stimuli of the same color, for both colors (Fig. 2C, top row). Interestingly, this effect was lost in intermediate and peripheral channels for blue deviants but robustly maintained for green deviants (Fig. 2C, middle and bottom rows). Blue and green stimuli have previously been shown to evoke different responses at the level of photoreceptors that project to distinct medulla neurons in the fly optic lobes (Yamaguchi et al., 2010), which could explain the differences in SSVEPs observed here. To confirm that our method of sampling channels (central, intermediate, peripheral; see Materials and Methods) was accurately representative of what individual channels were showing, we also re-analyzed the preceding experiment across a larger pool of channels, focused around the central and intermediate areas. In this, we found the same pattern of results: decreased responsiveness to deviants for only the five most central recording sites if deviants were blue, but across all sampled recording sites for green deviants (Fig. S1). We therefore continued to sample from the three distinct locations, along with counterbalancing of our visual experiments to ensure stimulus color did not confound our interpretations.

### The central brain is insensitive to carrier effects

**Fig. 3.**
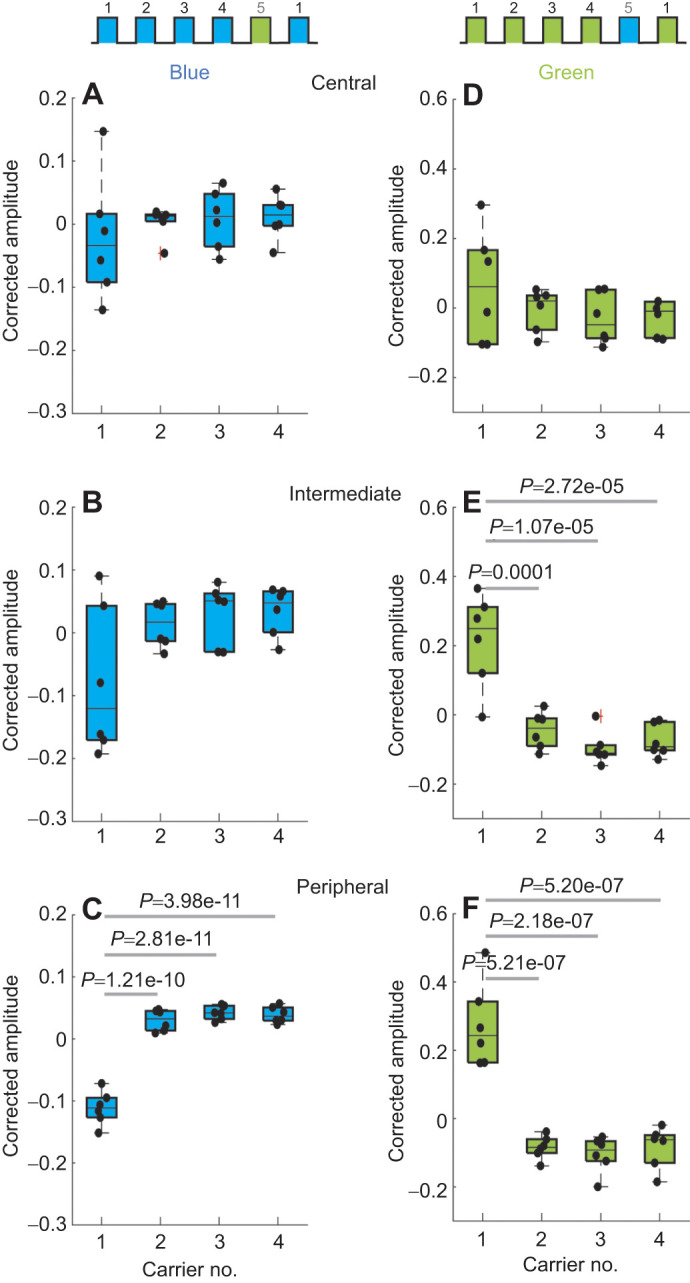
**The central brain is insensitive to carrier effects.** (A–C) Comparison of corrected amplitude for the first to fourth blue carrier after a green deviant, across central (A), intermediate (B) and peripheral (C) channels. Significance between groups is indicated with a gray bar and *P*-value, based on one-way ANOVA with Bonferroni correction (*N*=8). (D–F) As for A–C, for green carriers after a blue deviant, across central (D), intermediate (E) and peripheral (F) channels.

Not all carrier stimuli within a color were equal in the context of our paradigm. While their luminosity and hue were identical, their history was different (e.g. carriers at position 4 and 6 in Fig. 2A, middle and bottom rows). Thus, the carrier at position 6 can also be seen as deviant-like (a nature we exploited for appropriate comparison with the deviant, earlier). When we looked at these carrier orders specifically (renumbered as 1–4; Fig. 3) we found that the first carrier following a deviant (carrier 1) evoked a significantly different response from the other carriers that followed (carriers 2–4) (Fig. 3A–F). Colors had opposite effects in the optic lobe channels: blue carrier 1 was significantly smaller than the subsequent three blue carriers, while green carrier 1 was significantly larger than the three subsequent green carriers (Fig. 3C,F). This suggests a color change effect, which might be expected considering distinct color-processing pathways in the optic lobes of the *Drosophila* visual system (Paulk et al., 2013a; Yamaguchi et al., 2010). This effect persisted in the intermediate channels, although it was only significant for green carriers (Fig. 3B,E). Interestingly, the carrier effect was lost in the central channels (Fig. 3A,D). This suggests that SSVEPs recorded in the central fly brain are tracking stimulus probability rather than changes in stimulus color, which is consistent with our preceding observations (Fig. 2C).

### Stimulus probability rather than uncertainty determines SSVEP response amplitude

**Fig. 4.**
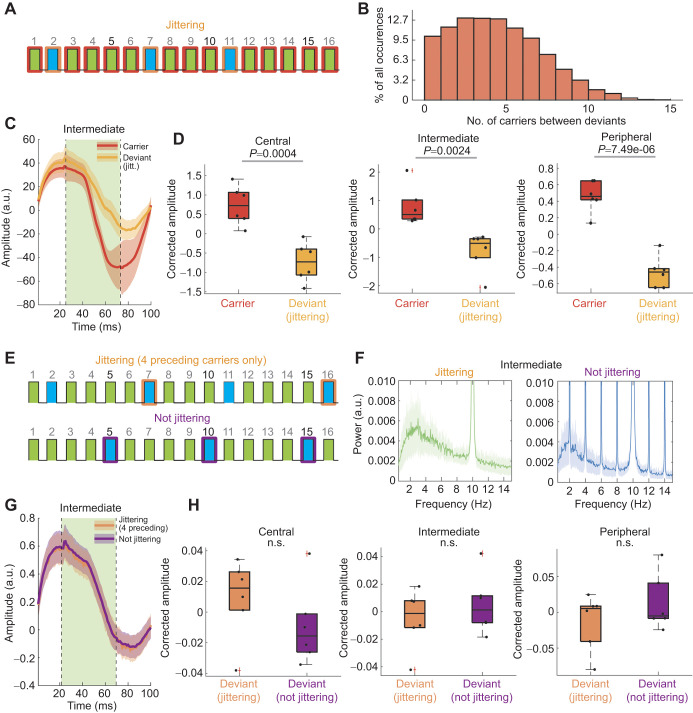
**Stimulus probability rather than uncertainty determines LFP response amplitude.** (A) Example stimulus schema for a block where the position of the deviant in sequence was ‘jittered’ according to a normal distribution. Orange-outlined boxes indicate example deviant positions, while red-outlined boxes indicate carriers used for comparison. (B) Calculated actual distribution of deviants in sequence (*N*=6). (C) Average LFPs across individuals for carriers of any position (red) versus deviants that appeared in jittered sequence positions (orange). Colors are combined here, such that carriers could have been either green or blue, and similarly for deviants. (D) Comparison of corrected amplitude values for carrier and deviant conditions mentioned above, across central, intermediate and peripheral channels. Dots each represent an individual fly average (*N*=6). Box plots show median, upper and lower quartiles and 1.5× interquartile range. *P*-values represent one-way ANOVA with Bonferroni correction. (E) Example schematized stimulus sequences for a ‘jittering’ and ‘not-jittering’ block. Colored boxes indicate the selection of a deviant stimulus event that happened to be preceded by four carrier events (orange outline), as well as a non-jittering deviant that would always be preceded by four carrier events (purple outline). (F) Power spectrum of LFP activity in intermediate channels during jittering (left) and not-jittering (right) trials, normalized and averaged across individuals. F1 carrier frequency can be observed at 10 Hz, while F2 deviant frequency peaks at 2 Hz. (G,H) As for C and D, but only comparing jittering (orange) and non-jittering (purple) deviants both preceded by four carrier events (one-way ANOVA with Bonferroni correction; *N*=6).

In the preceding paradigm, both the deviant and carrier stimuli were equally predictable; the deviant occurred after exactly four carriers. We next questioned whether decreasing the predictability of the deviant might alter how the fly brain responds to it. To test this, we jittered the position of the differently colored deviant stimulus such that it could appear after different numbers of carrier stimuli (Fig. 4A), while on average still occurring at 2 Hz compared with the 10 Hz carrier (Fig. 4B). All experiments were counterbalanced like before so that comparisons could be made within a color (i.e. green jittering deviant compared with a green carrier, blue jittering deviant compared with a blue carrier). We found that, like its invariant counterpart, a jittering deviant also evoked smaller responses compared with carrier SSVEP amplitudes (Fig. 4C), and the combined data were significant across the fly brain (Fig. 4D). To further investigate whether predictability affected SSVEP amplitude, we specifically examined only the subset of jittering deviants that happened to occur after exactly four carriers (Fig. 4E), thus providing a physically identical comparison for the non-jittering experiments presented in Fig. 2. A spectral analysis of the visual stimulus showed how different the context was for these two different paradigms (Fig. 4F). Nevertheless, when restricted to comparing identical physical histories (i.e. blue deviant preceded by four green carriers), we found no significant difference between a jittering and a non-jittering deviant (Fig. 4G), but noted a trend toward a difference in the central brain (Fig. 4H).

### Spontaneous sleep decreases the brain response to deviants specifically

**Fig. 5.**
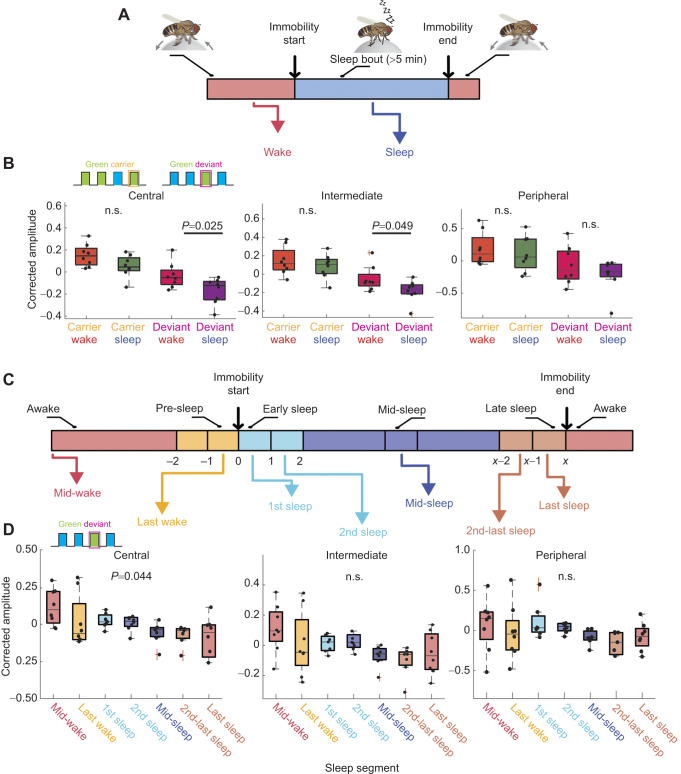
**Spontaneous sleep decreases the brain response to deviants specifically.** (A) Schema illustrating the conversion of locomotive activity data into wake and sleep bout identification. (B) Corrected amplitude values for green carrier and deviant responses during wake and sleep. Dots represent individual fly data (*N*=8). Box plots show median, upper and lower quartiles and 1.5× interquartile range. Comparisons were conducted only within group (e.g. carrier wake versus carrier sleep) and represent one-way ANOVA with Bonferroni correction. (C) Schema separating wake and sleep bouts into segments based on time since sleep onset (Jagannathan et al., 2024; Yap et al., 2017). Each segment represents a particular time period either before/after the onset of immobility or during sleep. (D) Corrected amplitude values for green deviant stimulus responses during the time segments listed in C. Dots represent individual fly data (*N*=8). Box plots show median, upper and lower quartiles and 1.5× interquartile range. *P*-values listed represent the overall significance of one-way ANOVA with Bonferroni correction.

In our earlier experiments, we found that spontaneous sleep had no effect on the amplitude of the SSVEP response to 10 Hz carrier stimuli presented throughout the night (Fig. 1). Knowing that the waking fly brain's response to deviant stimuli is different from its response to carriers, we next examined whether these were processed differently during spontaneous sleep compared with wake. As we observed the same result with deviants irrespective of whether they were jittering or predictable, we focused on our original design where deviants occurred at exactly 2 Hz (non-jittering) within a 10 Hz carrier of a different color. Flies were exposed to these stimuli during long-term multichannel recordings as before (these were the same overnight flies as in Fig. 1, which were also exposed to 20 s packets of only carrier stimuli), and sleep was identified as periods of quiescence lasting 5 min or more under these conditions (Fig. 5A). As before, we only compared effects within a color (e.g. green deviants to green carrier 1). We found that sleep significantly decreased the SSVEP response to deviant stimuli, while responses to carrier stimuli were still not significantly affected by behavioral state (consistent with Fig. 1 results; Fig. 5B). Interestingly, the effect of sleep on deviant responses was only significant in the central and intermediate brain channels and was not significant in the peripheral (outer optic lobe) channels. Together, these findings indicate that processing of monotonic stimuli (i.e. carriers) is unaffected by sleep throughout the fly brain, while processing of low probability (deviant) stimuli is affected by sleep, in the more central regions of the fly brain. The effect of sleep is related to stimulus probability rather than color, because our counterbalanced analysis ensured that the immediate stimulus history for both the compared carriers and deviants was always a transition between colors (see Materials and Methods).

### Spontaneous sleep stages have different effects on the response to deviant visual stimuli

In a recent study using the same multichannel brain recording preparation, we identified distinct stages of sleep based on classification of electrophysiological activity as well as on microbehaviors (Jagannathan et al., 2024). We therefore questioned whether the attenuated response to deviant stimuli was characteristic of any specific phase of sleep. To address this, we partitioned all identified sleep epochs (>5 min) into five segments (Fig. 5C): the first minute of sleep (1st sleep), the second minute of sleep (2nd sleep), the middle minute of sleep (mid-sleep; variable in position across bouts of different durations), the second to last minute of sleep (2nd-last sleep) and the last minute before awakening (last sleep). The randomized distribution of our visual paradigm throughout the night ensured that all counterbalanced blue/green presentations occurred multiple times for each sleep segment, for each fly (see Materials and Methods). For comparison, we performed the same analyses for two wake segments: data taken from the middle minute of a preceding wake epoch (mid-wake) and data taken from the last minute of preceding wakefulness (last wake), when flies were still moving (Fig. 5C). We found that only recordings taken from the central brain revealed significant differences among this segmented data (Fig. 5D), but with no specific sleep epoch significantly different on its own compared with other epochs.

**Fig. 6.**
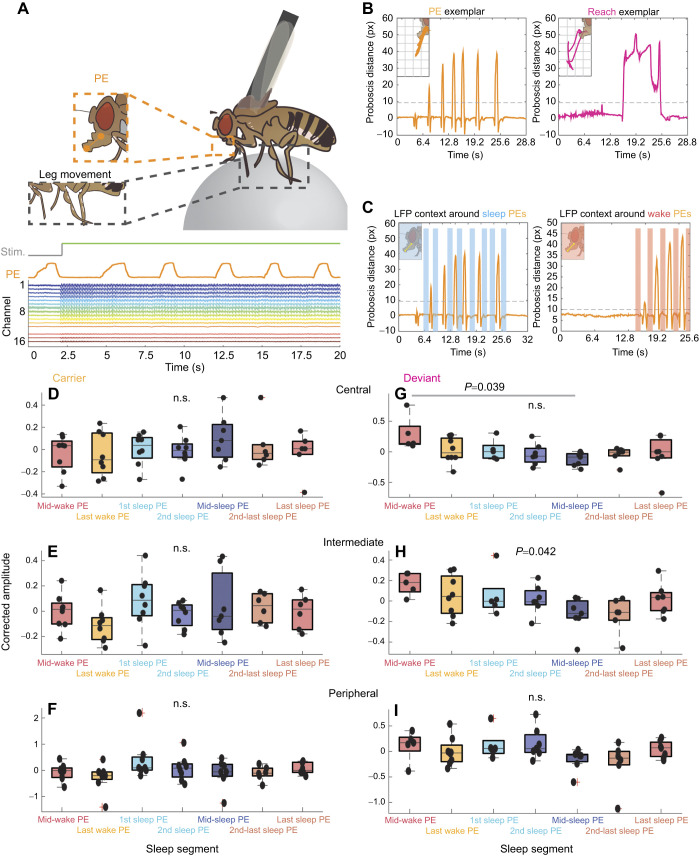
**Decreased response to deviant stimuli during deep sleep specifically.** (A) Top: schema of a tethered fly, focusing on the tracking of proboscis extensions (PE, orange) and locomotive activity via leg movement (black). Bottom: LFP activity (colored traces) in an example fly, during a spell of proboscis extensions (orange trace). Minimal proboscis extension artifact is evident in the brain recordings. The presence of visual stimuli during this period is indicated above (gray/green trace). (B) Left: exemplar proboscis extension distance tracked from resting position (orange) during a proboscis extension spell. Inset, a trace of the position of the proboscis in 2D space during the event. Right: as for left but for a ‘reach’ event, showcasing the longer time course and singular nature. (C) Left: exemplar proboscis extension spell during a sleep bout, with inclusion of shading to show portions of time when LFP activity was sampled. Right: as for left, for a proboscis extension spell occurring during a wake bout. (D–F) Corrected amplitude values for green carrier events occurring during proboscis extensions of specific sleep segments, across different brain regions. Significance calculated via one-way ANOVA with Bonferroni correction. (G–I) As for D–F, for green deviant events. For D–I, dots represent individuals (*N*=8). Box plots show median, upper and lower quartiles and 1.5× interquartile range. Significance comparisons between groups are represented with gray lines while overall significance is presented purely as text (one-way ANOVA with Bonferroni correction.

In the preceding analyses, sleep stages were inferred by their timing following the beginning of a spontaneous sleep bout. While this has been indicative of when active or quiet sleep stages might occur (Yap et al., 2017), we sought an alternative behavioral approach to disambiguate distinct sleep stages in our data. In a recent study, we found that flies displayed more rhythmic proboscis extensions during their deep sleep stage (Jagannathan et al., 2024), which supports an earlier finding (van Alphen et al., 2021). We therefore wondered whether responses were affected during the deep sleep epochs characterized by bursts of rhythmic proboscis extensions. As rhythmic proboscis extensions also sometimes occur during wake in this preparation (Jagannathan et al., 2024), this provided an ideal comparison to determine whether behavioral state (i.e. deep sleep) or just the behavior itself (i.e. proboscis extension) governed the weakened response to deviant visual stimuli. As previously (Jagannathan et al., 2024), we employed machine learning approaches to identify proboscis extensions in our overnight recordings of flies exposed to visual stimuli, while also tracking leg movements to monitor sleep (Fig. 6A, top). We identified all proboscis extension activity that qualified as rhythmic and aligned them in time with our multichannel electrophysiological activity (Fig. 6A, bottom). Closer examination of proboscis activity revealed two kinds of movements, either rhythmic (‘proboscis extensions’) or individual, questing-like ‘reaches’, which primarily happened during wake (Fig. 6B). Given their purported relevance for sleep functions (van Alphen et al., 2021) we only examined proboscis extensions. As proboscis extensions themselves affect fly brain activity (Jagannathan et al., 2024), we only examined SSVEPs that were in between the rhythmic proboscis extension events (Fig. 6C), to determine whether the proboscis extension ‘context’ (during wake versus sleep) affected SSVEP amplitude (see Materials and Methods). We identified rhythmic proboscis extension events in all of our data, allowing us to partition these into the same temporal segments as outlined above. Consistent with our results so far, sleep had no effect at all on how carrier stimuli were processed (Fig. 6D–F). In contrast, deviant stimuli were sensitive to sleep quality (Fig. 6G–I). Interestingly, only proboscis extensions occurring during mid-sleep epochs (when flies are in deep sleep; Jagannathan et al., 2024; van Alphen et al., 2021) were associated with significantly decreased responsiveness to deviant stimuli in the central brain (Fig. 6G), although other sleep epochs showed a trend in the same direction. This effect was not seen in the optic lobe recordings, while data taken from intermediate channels seemed midway between those from the other two channel groups (Fig. 6H,I). This suggests that during the deepest phase of sleep, when flies typically display the most rhythmic bursts of proboscis extensions (Jagannathan et al., 2024), the central fly brain is significantly less responsive to low-probability stimuli but equally responsive to high-probability stimuli. During equivalent wake epochs, these differences disappear.

## DISCUSSION

Convincing evidence that *Drosophila* flies really sleep was first provided by showing that flies had increased arousal thresholds to mechanical stimuli if they had been inactive for 5 min or more (Hendricks et al., 2000; Shaw et al., 2000). Subsequent experiments also using mechanical stimuli confirmed 5 min of immobility as an accurate threshold for studying sleep in this model (Huber et al., 2004; van Alphen et al., 2013), although recent studies have questioned whether sleep quality is adequately addressed by this proxy (Chowdhury et al., 2023; Tainton-Heap et al., 2021). Other recent studies have used olfactory and air-puff stimuli to assess sleep quality and intensity in *Drosophila* (French et al., 2021; Joyce et al., 2024). Here, we used visual stimuli to investigate sensory processing during sleep, and we focused on LFP responses across the fly brain rather than behavior. We found that repetitive visual stimuli are processed similarly during sleep and wake, but lowering the probability of a stimulus made it less detectable in the central brain during sleep. This indicates that visual stimuli are gated during sleep in *Drosophila*, similar to what has been shown in the olfactory modality (French et al., 2021). This effect reached a nadir during the deeper stages of sleep, when flies displayed rhythmic proboscis extensions. This confirms for another sensory modality (vision) that flies are indeed sleeping after 5 min of immobility. This finding also has relevance for uncovering sleep functions: deviant stimuli are typically surprising, and one proposed function of sleep is to optimize how prediction errors are processed (Van De Poll and van Swinderen, 2021). It is therefore possible that altered processing of deviant stimuli during sleep also accomplishes a homeostatic function. In humans, REM quantity has been linked to altered waking predictive processing (Barsky et al., 2015). While a link between visual experience and sleep has not been well investigated in flies, one study found that exposing flies to more complex visual stimuli (e.g. a movie sequence compared with a scrambled version) increases their subsequent sleep need (Kirszenblat et al., 2019), and another study found that sleep deprivation impairs visual responsiveness in flies (Kirszenblat et al., 2018). There is therefore already good evidence that sleep modulates visual processing in *Drosophila*. Interestingly, in our data, the effect of sleep on visual predictive processing was only evident in the central brain recordings, despite the fact that the entire fly brain – even the optic lobes – was found to display distinct sleep features in multichannel LFP recordings (Jagannathan et al., 2024). This suggests that any mechanism suppressing the detection of deviant visual stimuli likely resides in the vicinity of the central brain, while the ‘sleeping’ optic lobes are still able to process these deviant stimuli in the same way as during waking. This is consistent with previous studies uncovering distinct sleep-related features in the central brain LFP activity (Yap et al., 2017).

One surprising finding from our study was that deviant visual stimuli evoked smaller LFP responses in the fly brain compared with high-probability carrier stimuli. In mammalian studies, deviant stimuli typically evoke larger responses in LFP or EEG recordings (Shiramatsu and Takahashi, 2021). Indeed, such amplified EEG effects have formed a foundation for studying predictive processing in humans and other mammals, whereby unexpected events evoke a comparatively larger ‘dip’ in brain recordings, termed ‘mismatch negativity’ (MMN; Näätänen, 1990). In our recordings, we found nothing resembling MMN. Rather, we found the opposite: a decreased response even to a properly unpredictable stimulus, and a further attenuated response to deviant stimuli during sleep – but only in the central brain. How might this discrepancy with similar readouts in other animals be explained? One explanation lies in our unique recording preparation. In our multichannel recording setup, the linear array of 16 recording sites was oriented along a specific plane, facing the back of the fly's head (see Fig. 1B). Field potential electrodes only record what they are allowed to detect, namely parallel electrical fields lying in a specific orientation so as to reveal a current and thus a voltage differential. It is therefore possible that other electrical fields orthogonal to the detected field are increasing in amplitude with certain stimuli (e.g. deviants) and thereby cancelling to some extent the fields we are measuring in the current experimental design. This is a known drawback of electrophysiology: orthogonal fields cancel out, and even a LFP flatline does not necessarily indicate a lack of neural activity (Buzsáki et al., 2012). In our study, we detected an effect of deviant stimuli, which necessarily means distinct processing for those stimuli – irrespective of how that processing is manifested in LFP activity. Future studies, for example using sharp recordings in the central brain rather than linear probes across the brain, should reveal whether deviant visual stimuli always evoke smaller LFP responses in *Drosophila*, or whether this might indeed just be a feature of the orientation of the multichannel recording electrodes used in this study.

Another surprising finding from our study was that making the deviant less predictable did not significantly alter the LFP response, compared with a predictable deviant with the same historical features. One might have expected an unpredictable deviant to be more surprising, therefore evoking an even more attenuated version of the deviant response. Rather, if anything, there was trend to a larger response in the unpredictable (or ‘jittering’) deviant in the central brain (Fig. 4D). This again calls for the employment of a variety of approaches for studying predictive processing in the central fly brain, now that we have shown that identical visual stimuli are not all processed equally depending on behavioral state and history. Other *Drosophila* studies have shown that visual responses depend on waking behavioral states. For example, visually responsive neurons in the optic lobes receive self-motion cues that assist with suppression of optomotor-related effects (Kim et al., 2015) which may in turn interact with neuronal activity in a state-dependent manner to dynamically modulate processes such as navigation and locomotion (Fujiwara et al., 2022). For the purposes of investigating predictive processing following the paradigms presented in the current study, performing experiments with altered stimulus frequencies or predictabilities could help to uncover the underlying mechanisms, as has been done for human psychophysics experiments (Gökaydin et al., 2015). Additionally, more focused LFP recording preparations (Grabowska et al., 2020) as well as calcium imaging (Tainton-Heap et al., 2021; Troup et al., 2023), coupled with transient manipulation of visual processing circuits in the central brain, should further elucidate the nature of the variable deviant responses we have uncovered in this study.

## Supplementary Material

10.1242/jexbio.250165_sup1 Supplementary information
