# Supplementary material for: Long-term multichannel recordings in Drosophila flies reveal altered predictive processing during sleep compared with wake
Source: J Exp Biol. 2025 Jun 3;228(11):jeb250165. doi: 10.1242/jeb.250165 (PMC12188248; doi:10.1242/jeb.250165)
Supplement: Supplementary information [file jexbio-228-250165-s1.pdf]

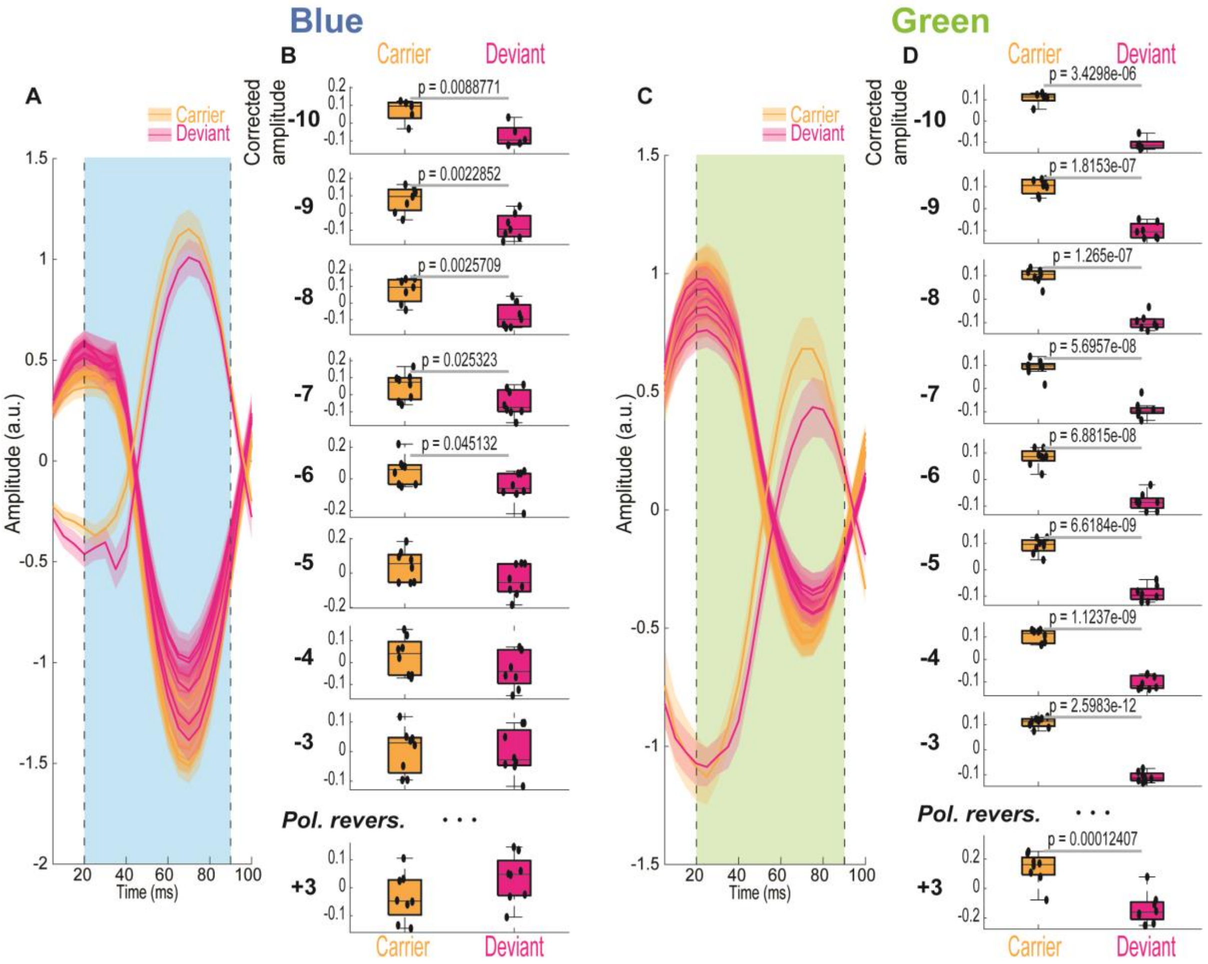

Fig. S1. Neighboring LFP channels exhibit similar responsiveness to carrier and deviant stimuli. **A.** Averaged LFP responses to blue carrier (orange shading) and deviant (pink shading) stimuli. Stimulus timecourse is shown with blue shading and dashed vertical lines. Data is shown for 8 individual channels, from an average of N=8 individuals. **B.** Corrected amplitudes for the carrier (orange) and deviant (pink) LFPs shown in A, separated by individual channel. Each channel is defined according to its position relative to the polarity reversal for an individual. P-values where indicated represent one-way ANOVA with Bonferroni correction (N=8). **C,D.** As with A,B, for green stimuli.
